# Complete mitochondrial genome of melon fly, *Zeugodacus cucurbitae* (Diptera:Tephritidae) from Kunming, Southwest China and the phylogeny within subfamily Dacinae

**DOI:** 10.1080/23802359.2020.1790318

**Published:** 2020-07-15

**Authors:** Xiao-Hong Zhou, Jian-Hong Liu, Qiu-Lang Zhang, Xiao-Shuang Wan, Da-Ying Fu, Xu-Bo Wang, Wen-Li Dan, Mei-Jun Yang

**Affiliations:** a Key Laboratory of Forest Disaster Warning and Control of Yunnan Province, Southwest Forestry University, Kunming, China; b College of Life Science, Shangrao Normal University, Shangrao, China

**Keywords:** *Zeugodacus tau*, quarantine pest, mitogenome, molecular phylogeny

## Abstract

The melon fly, *Zeugodacus cucurbitae* (Diptera:Tephritidae) is an important invasive pest and distributed throughout tropical, subtropical countries and areas. In this study, we report the complete mitochondrial genome of the fly with 15, 685 base pair in length, which includes 37 genes (the large and small ribosomal RNA subunits, 22 transfer RNA genes, 13 genes encoding mitochondrial proteins) and a non-coding A + T-rich control region. Molecular phylogeny indicated that there was a high bootstrap value supported among *Z. cucurbitae* and *Z. tau* belonging to the *Z. tau* complex.

Melon fly, *Zeugodacus cucurbitae* (Diptera: Tephritidae), is an important invasive pest in fruits and vegetables (Matthew and Jang 2010), infesting primarily the fruit and flower of Cucurbitaceae (Vargas et al. 2015). Although native to India, it is now widely distributed in tropical, sub-tropical and temperate regions worldwide (Dhillon et al. 2005; Virgilio et al. 2010). In this study, we reported the complete mitogenome of *Zeugodacus cucurbitae* from Kunming, Southwest China. The result would be greatly helpful for phylogenetics, population genetics, and species identification.

The adult male flies were caught from Kunming (25.05°N, 102.76°E), Southwest China, on 12 July 2018. Specimen was deposited in the museum of Southwest Forestry University (Voucher ZCKM20180712), Kunming, China. Whole genomic DNA was extracted from adult fly following the manufacturer’s instruction in the DNeasy Blood and Tissue kit (Qiagen, Hilden, Germany) and then sequenced and assembled using Illumina’s HiSeq2000 platform (Illumina, San Diego, CA). The sequence was preliminarily aligned within the CLUSTAL X program in BioEdit software. Protein-coding genes (PCGs), rRNA genes were predicted by using MITOS tools (Bernt et al. 2013), and tRNA was done through tRNAscan-SE (Lowe and Chan 2016).

The complete mitogenome of *Z. cucurbitae* is 15,685 bp in length (GenBank MH900082), containing 13 PCGs, 22 transfer RNA genes, 2 ribosomal RNA genes, and a major non-coding region known as the CR (control region). The base composition is 38.49% for A, 34.34% for T, 16.67% for C, and 10.50% for G. A total of 23 genes, including 9 PCGs(NAD2-3, NAD6, COX1-3, CYTB, ATP6, and ATP8) and 14 tRNAs, were distributed on the J-strand of mitogenome, while the remaining 14 genes, including 4 PCGs (NAD1, NAD4, NAD4l, and NAD5), 8 tRNAs, and 2 rRNAs (16S rRNA and 12S rRNA), are located on the N-strand. The genes arrangement is conservative and identical to the most common type of the ancestor insect *Drosophila yakuba* (Clary and Wolstenholme 1985).

A phylogenetic tree was established based on complete mitogenome of *Z. cucurbitae* and other 20 species published Tephritidae species having Refseqs in NCBI by BI method in MrBayes version 3.2.6 (Huelsenbeck and Ronquist 2001; Dereeper et al. 2010). The result indicated that there was 100% bootstrap supported among *Z. cucurbitae* and *Z. tau,* which belong to *Z. tau* complex (Figure 1). Furthermore, four clades were correctly identified as assigned *Bactrocera*, *Zeugoacus*, *Dacus* and Ceratitidini with high bootstrap confidence, while *Zeugoacus* and *Dacus* form a sister group (Figure 1). In conclusion, the mitochondrial genome of *Z. cucurbitae* educed in the present study can provide essential DNA molecular data for further phylogenetic and evolutionary analysis and it will facilitate the development of new DNA markers for species diagnosis, therefore it will be helpful for improving accurate detection of quarantine species.

**Figure 1.**
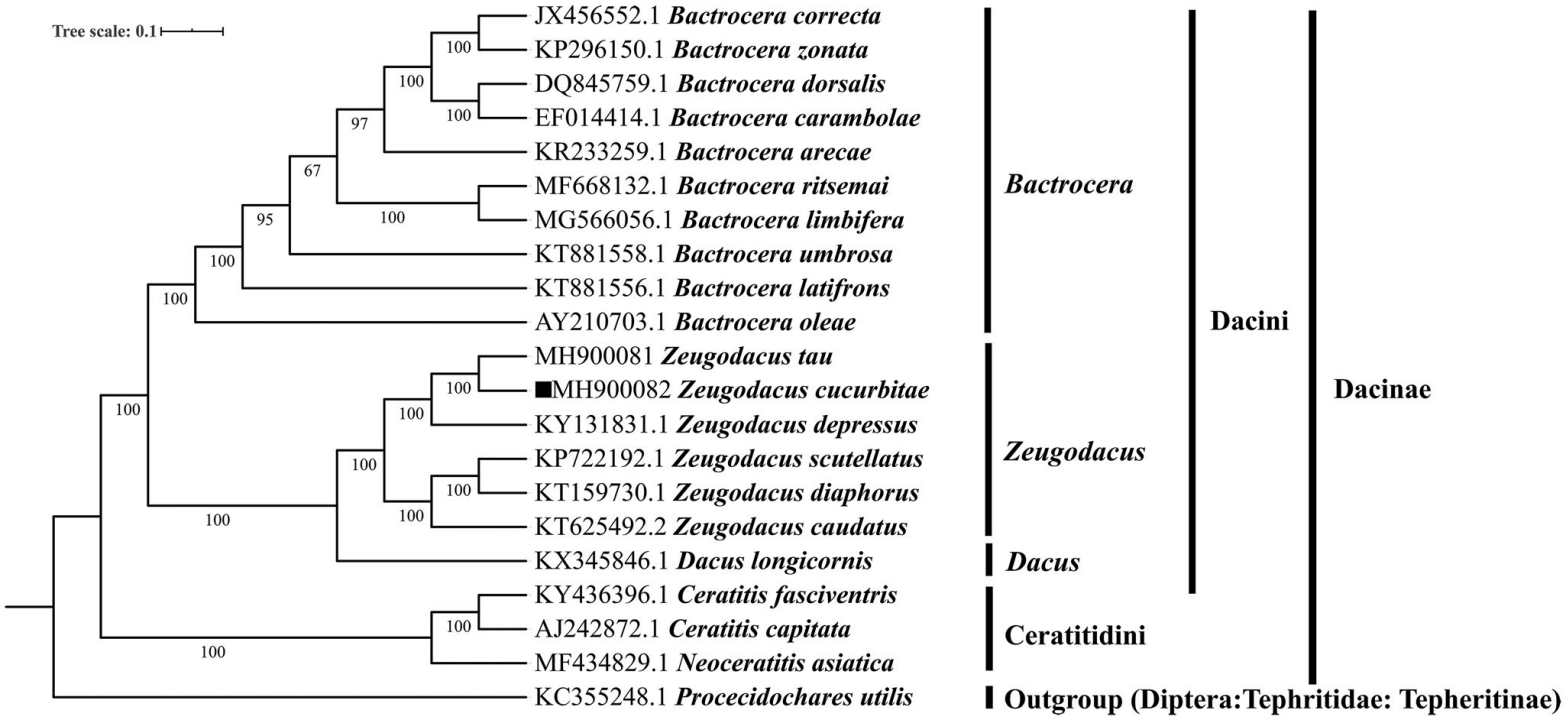
Molecular phylogeny for *Zeugodacus cucurbitae* and the related species in subfamily Dacinae based on complete mitogenome. Tree was constructed by Bayesian Inference (BI) method. Genbank accession numbers lie before the scientific name of species. The position of *Z. cucurbitae* is marked with solid square shape.

## Data Availability

The data that support the findings of this study are openly available in GenBank of NCBI at https://www.ncbi.nlm.nih.gov, reference number MH900082.
